# Solving the plastic dilemma: the fungal and bacterial biodegradability of polyurethanes

**DOI:** 10.1007/s11274-023-03558-8

**Published:** 2023-03-17

**Authors:** Parth Bhavsar, Mrinal Bhave, Hayden K. Webb

**Affiliations:** Department of Chemistry and Biotechnology, Swinburne University of Technology, John St, Hawthorn, VIC 3122 Australia

**Keywords:** Biodegradable polymers, Biodegradation, Plastic disposal, Polyurethane, Recycling

## Abstract

Polyurethane (PU) is a plastic polymer which, due to its various desirable characteristics, has been applied extensively in domestic, industrial and medical fields for the past 50 years. Subsequently, an increasing amount of PU waste is generated annually. PU, like many other plastics, is highly resistant to degradation and is a substantial threat to our environment. Currently PU wastes are handled through conventional disposal techniques such as landfill, incineration and recycling. Due to the many drawbacks of these techniques, a ‘greener’ alternative is necessary, and biodegradation appears to be the most promising option. Biodegradation has the potential to completely mineralise plastic waste or recover the input materials and better enable recycling. There are hurdles to overcome however, primarily the efficiency of the process and the presence of waste plastics with inherently different chemical structures. This review will focus on polyurethanes and their biodegradation, outlining the difficulty of degrading different versions of the same material and strategies for achieving more efficient biodegradation.

**Supplementary Information:**

The online version contains supplementary material available at 10.1007/s11274-023-03558-8.

## Introduction

Polyurethane (PU) has had extensive applications in the domestic, industrial and medical fields for the past 50 years. Its intrinsic structure gives it high tensile strength, high resistance to hydrolysis and a high melting point (Ibrahim et al. 2009; Loredo-Treviño et al. 2012). It is synthetically prepared and used in adhesives, foams, food-grade coatings, insulators, tyres, sponges and many more products (Matsumura et al. 2006). The global PU production in 2016 was around 22.3 million tonnes with a growth rate of 4.0% per annum (Austin and Hicks 2017). In Europe alone polyurethane contributed to nearly 7.7% of the total plastic demand; around 4 million tonnes in 2017 (PlasticsEurope 2018). In Australia it is estimated that approximately 2.5 million tons of plastic waste were generated in the year 2016–17, out of which only 12% was recycled and 87% was dumped in landfills, where it will remain for many years to come (Pickin et al. 2018). If the world continues producing plastic waste at the current rate, then over 12000Mt will accumulate by the year 2050 (Geyer et al. 2017). This review will focus on polyurethanes and their biodegradation, in order to define in greater detail this portion of the larger issue of the environmental hazards of plastic.

## Environmental impact of polyurethanes

Increase in PU demand over the last 50 years has led to a major bottleneck in determining an efficient and clean method for its disposal. Subsequently, it accumulates in the environment, both terrestrial and aquatic. Due to their low density, most plastics tend to float, and so are carried by floods, high tides etc. into waterways and finally reach the oceans, where they cause irreversible damage to that ecosystem (Derraik 2002). Various surveys have revealed that out of the five major Ocean gyres, the South Pacific subtropical gyre and the eastern side of the North Pacific Ocean gyre are the hotspots for the accumulation of plastic debris (Kaiser 2010; Eriksen et al. 2013; Law et al. 2014). The lack of suitable waste management practices in many coastal countries is directly related to this problem (Jambeck et al. 2015). Almost 90% of the oceanic plastic waste comes from just ten rivers which originate from Asia and Africa (Schmidt et al. 2017), and studies have shown that at least 690 marine species are directly affected by plastic debris, be it through ingestion, entanglement, and/or smothering (Gall and Thompson 2015).

A major concern is the formation of microplastics from these debris. Plastic waste undergoes limited degradation in the environment and fragments into microplastics (Rillig 2012; Su et al. 2019). Due to their minute size (< 5 mm), they easily invade the local food chain and bioaccumulate. Microplastic contamination has reached a stage where they have even been found in the placentas of unborn babies (Ragusa et al. 2021). Microplastics also have a tendency to absorb organic pollutants from the surrounding environment, becoming toxic (Rillig 2012; Zhu et al. 2019). Persistent organic pollutants (POPs) such as pesticides, polycyclic aromatic hydrocarbons (PAHs) and polychlorinated biphenyls (PCBs) are of particular concern (Rios et al. 2010; Fisner et al. 2013).

## Polyurethane—production and properties

Polyurethane was first produced in the 1930s by Otto Bayer and his team in pursuit of a new polymer to compete with Nylon 6,6. Initially, they produced polyurea by reacting diisocyanates with either aliphatic or aromatic diamines, but these polymers were too hydrophilic for their use as plastics. By exchanging diamines for diols, they were able to produce polyurethanes, which proved much more useful. One polymer produced by reacting 1,4-butanediol and hexamethylene diisocyanate (HDI) had very similar properties to Nylon, but with better electrical and mechanical stability, and so quickly became widely popular (Heath and Cooper 2013).

### Properties and characteristics

Polyurethanes are segmented polymers made up of crystalline and amorphous regions. The crystalline segments provide the polymer with strength, while the amorphous segments impart flexibility. The hardness and the elasticity of polyurethane can be customised by controlling the ratio of each, making polyurethanes suitable for a range of products, from rigid foams to elastomer fibres (Shelke et al. 2014). Due to this versatility, polyurethanes are applied as dispersions, coatings, adhesives, foams, fibres and more. Over the last two decades, due to their biocompatibility they have been useful in biomedical fields, such as in the making of artificial pacemakers, blood bags, catheters, insulators, grafts etc. (Heath and Cooper 2013). Table 1 lists some physical properties of some representative polyurethanes, demonstrating the versatility of this group of polymers.

**Table 1:** Physical properties of example polyurethane products, demonstrating the versatility of the polymer

PU types	Product (Manufacturer)	Density (g/cc)	Tensile strength (MPa)	Strain/elongation at break (%)	Abrasion resistance (mm^3^)	Shore hardness A/D	Melting point (°C)
Adhesive foam	Isomat PU-Foam Thermo (ISOMAT S.A)	0.018–0.020	0.07	20	ND	ND	ND
Polyester PU elastomer	Polyurethane D44 (QUADRIGA Dichtungs-GmbH)	1.24	49	570	29	80 A	ND
	Polyurethane D44 (QUADRIGA Dichtungs-GmbH)	1.25	48	600	32	85–90 A	ND
	Polyurethane D44 (QUADRIGA Dichtungs-GmbH)	1.24	46	550	30	67–77 A	ND
Aliphatic PU coating	Topcoat-PU 720 (ISOMAT S.A)	1.16	20.5	150	AR 0.5	57D	ND
Thermoplastic PU	Desmopan 192 (Covestro)	1.23	52	590	32	92 A	210–225
	Desmopan 385 S (Covestro)	1.2	53	630	36	85 A	210–230
	Ester type Addigy® (Covestro)	1.23	43	450	ND	60–64D	ND
Rigid PU	P1001 (Marco rubber)	ND	34.5	10 to 1580	ND	90 A	> 250

Data retrieved from manufacturers’ technical data sheets

*ND* No data available

### Polyurethane chemistry, synthesis and manufacturing

**Fig. 1:**
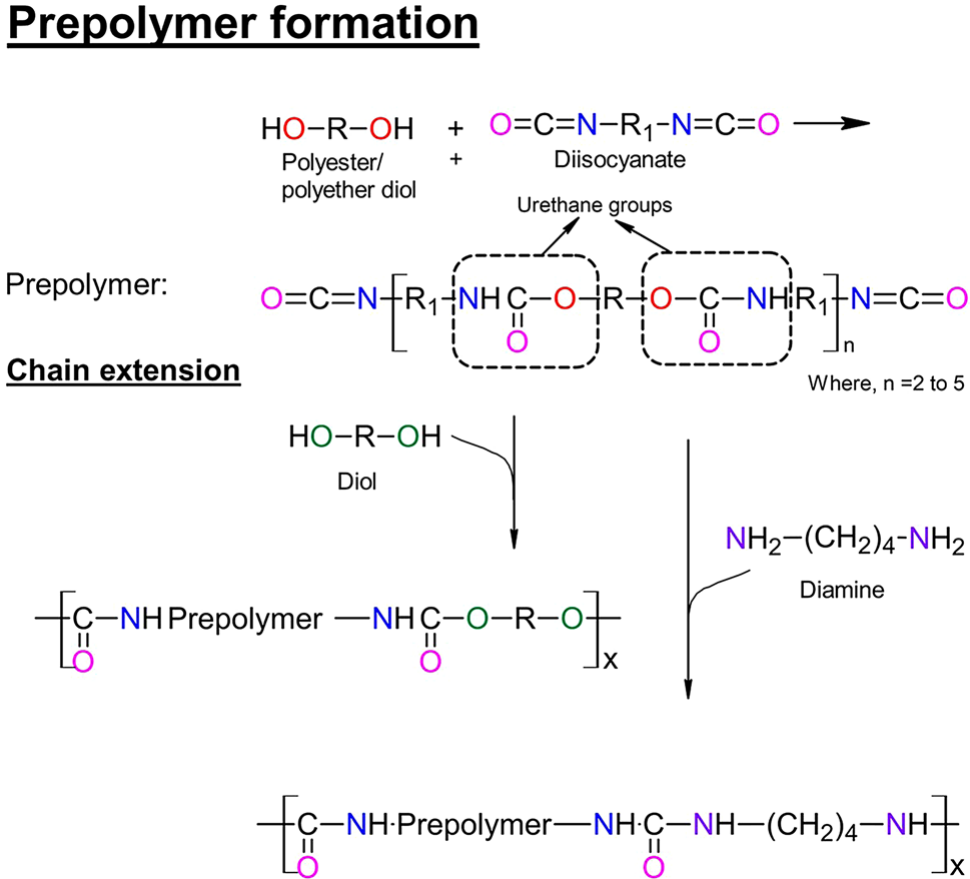
Schematics of typical polyurethane syntheses. A prepolymer is first produced by reaction between a diol and diisocyanate, forming regular urethane (NC(O)O) linkages. Chain extension is then performed on the addition of either additional diol or diamine. Modified from (Heath and Cooper 2013)

Polyurethanes are synthesised by reacting diisocyanates, polyglycols and chain extenders in a specific order and proportion depending on the type of polyurethane needed (Heath and Cooper 2013). They are produced generally by the exothermic reaction between the reactive hydroxyl groups of the polyglycols and the isocyanate groups of the diisocyanates present in excess (Shelke et al. 2014). This initially results in the formation of a prepolymer with regular urethane groups as shown in Fig. 1. This prepolymer is then reacted with chain extenders; commonly low molecular weight diols or diamines (Shelke et al. 2014). Some of the commonly used diisocyanates, diols and chain extenders are listed in Table 2.

**Table 2:** Common diisocyanates, diols and chain extenders used in the synthesis of PU

Precursor Component	Name	Structure
Diisocyanates	1,6- Hexamethylene diisocyanate (HDI)	
	2,4-Toluene diisocyanate (TDI)	
	4,4’-Methylene diphenyl diisocyanate (4,4’-MDI)	
Diols	Polycaprolactone diol	
	Polyether: Polypropylene glycol	
	Polyester: Polyethylene glycol adipate	
Chain Extenders	Diol: 1,4-butanediol	
	Diamine: ethylenediamine	

Structures drawn using ChemDraw Professional (version 20.1.0.110)

Different combinations of diisocyanates and diols give PU materials of varied characteristics. Polyglycols such as polyethers, polyesters, polycaprolactones and polycarbonates constitute the major fraction of polyurethanes (Mahajan and Gupta 2015). The polyglycol segments of polyurethane chains account for the amorphous sections of the bulk materials and so longer segments/higher content of polyglycols results in a softer PU with greater flexibility. The crystalline segments are mainly due to extensive hydrogen bonding between the urethane groups formed from the diisocyanates. Using shorter and/or lower content of polyglycols or using shorter diisocyanates during the synthesis of PU increases the frequency of urethane bonds and will result in a harder, more rigid PU (Young and Lovell 2011).

The ratio of the amorphous to crystalline segments and the specific polyglycols used will determine how susceptible a PU is to biodegradation. Softer (i.e. more amorphous) segments and polyester polyglycols are preferred for a more biodegradable PU (Kim and Kim 1998). Polyester PUs are more biodegradable due to the presence of hydrolysable ester moieties that are more prone to microbial enzyme attacks, and more amorphous chain packing makes these groups more accessible (Young and Lovell 2011; Mahajan and Gupta 2015; Kemona and Piotrowska 2020; Jin et al. 2021).

## Conventional disposal of polyurethane

Polyurethane products are commonly ‘single use’ items, and so are often discarded a short time after having fulfilled their intended purpose. There are three conventional methods employed to dispose of plastic waste and attempt to mitigate its accumulation. Each has its limitations, as outlined below, and alternative approaches such as large-scale biodegradation must be developed.

### Landfill

Landfill is the simple act of burying wastes and leaving them to their fate. Plastics, and especially polyurethane, are highly resistant to environmental degradation, and this is exacerbated by the limited exposure to sunlight and oxygen. Thus they persist for a very long time, and the land where these wastes are buried remains occupied for many years to come (Yang et al. 2012). Landfill is slow and occupies space that could otherwise be used for more productive purposes (e.g., farming, energy production). Another drawback is the slow and steady release of microplastics and other harmful chemicals into the surrounding soil. This obviously impacts the immediate environment, but the effects can spread when these materials leach off during rain and floods and/or are otherwise transported into nearby water bodies (Oehlmann et al. 2009; Teuten et al. 2009; Su et al. 2019). Because of this, disposal in landfill is not an efficient or future-friendly alternative (Hopewell et al. 2009). It is an ‘out of sight, out of mind’ approach to the problem.

### Incineration

By burning plastics some of the energy contained in the material can be recovered in the form of heat, which can then be converted to electricity or other mechanical force. It is estimated that burning a kilo of polyurethane waste yields a calorific value of around 7000 kcal/kg, which is equivalent to coal (Yang et al. 2012). Incinerating PU waste also reduces its solid volume by 99%, thus eliminating the need for huge land space associated with landfills. However, this method has a very big disadvantage of releasing a large number of air pollutants. Carbon dioxide (CO_2_), carbon monoxide (CO), hydrogen cyanide (HCN), hydrogen halides, oxides of nitrogen, isocyanates and more are released into the atmosphere (McKenna and Hull 2016). Air pollution control systems (APCs) can offset some of these pollutants, but not all incineration plants are capable of achieving good emission standards (Makarichi et al. 2018). Some of these compounds are chemical asphyxiant gases, like CO and HCN, and cause irreversible damage and death (Hartzell 1996). Hydrogen halides, isocyanates and oxides of nitrogen are irritant gases and cause a range of complex symptoms from skin irritation, tears, and pain in the chest to severe respiratory disruption. Carbon dioxide is of course one of the most powerful greenhouse gases and the primary cause of the greenhouse effect (McKenna and Hull 2016).

### Recycling

Polyurethanes are commonly recycled via chemical processing or mechanical processing (Yang et al. 2012). As the name suggests, chemical processing involves the use of chemicals to create useful raw materials from scrap PU (Yang et al. 2012). The polyurethane is depolymerised by chemolysis, generating end products dependent on the chemicals used and the PU composition. Chemical processing can be performed by glycolysis/alcoholysis (using low molecular weight alcohols), hydrolysis (water), aminolysis (alkanolamine) and phosphate ester methods (dimethyl phosphonate) (Xue et al. 1995; Troev et al. 2000; Yang et al. 2012). The end products are then purified and used as raw materials for producing new plastic. The process is however costly and tedious, has high safety requirements and can produce hazardous by-products.

Mechanical processing is somewhat more convenient and cheaper. Mechanical recycling of polyurethane is performed by grinding into granules, powder or flakes via mechanical force and using these in the production of new materials. The chemical structure of the scrap PU remains essentially the same and only the physical form changes. PU flakes can be re-bound with isocyanate or coated with binders and pressed under heat to produce new items. Granules or powder can be used as a filler material in making new parts, and granules can also be injection-moulded to make recycled PU materials. The scrap granules are exposed to high temperatures (~ 180 ℃) and high pressure (~ 350 bar), which allows the particles to melt and integrate/bind without using any additional binding agents (Scheirs 1998; Yang et al. 2012). All in all, while recycling overcomes the limitations of landfill and incineration, it is generally costly and not very efficient, and the physical integrity of the product decreases with every cycle. A comparison of the three standard disposal methods is presented in Table 3.

**Table 3:** Some of the advantages and disadvantages of conventional disposal methods

Method	Advantages	Disadvantages
Landfill	1. Simple to operate 2. Easy to maintain with minimum input.	1. Longer time to decompose. 2. The land is occupied for many years.
Incineration	1. Energy recovery 2. Well established technology. 3. Can accept almost all PUs.	1. Emission of poisonous gases. 2. Causes severe air pollution. 3. Hazardous to human health.
Physical recycling	1. Simple to operate with small equipment investments. 2. Much lower emission of pollutants. 3. Good efficiency	1. Not all PUs are compatible. 2. Diminished product performance and limited market use. 3. Very low profits.
Chemical recycling	1. Can obtain purified raw materials. 2. Raw materials used to generate new PUs. 3. New product’s performance stays the same.	1. Requires high temperature and pressure. 2. Complicated operation. 3. Equipment and maintenance cost very high. 4. Requires purification of the product before use. 5. Can produce dangerous by-products.

## Environmental degradation of polyurethane

**Fig. 2:**
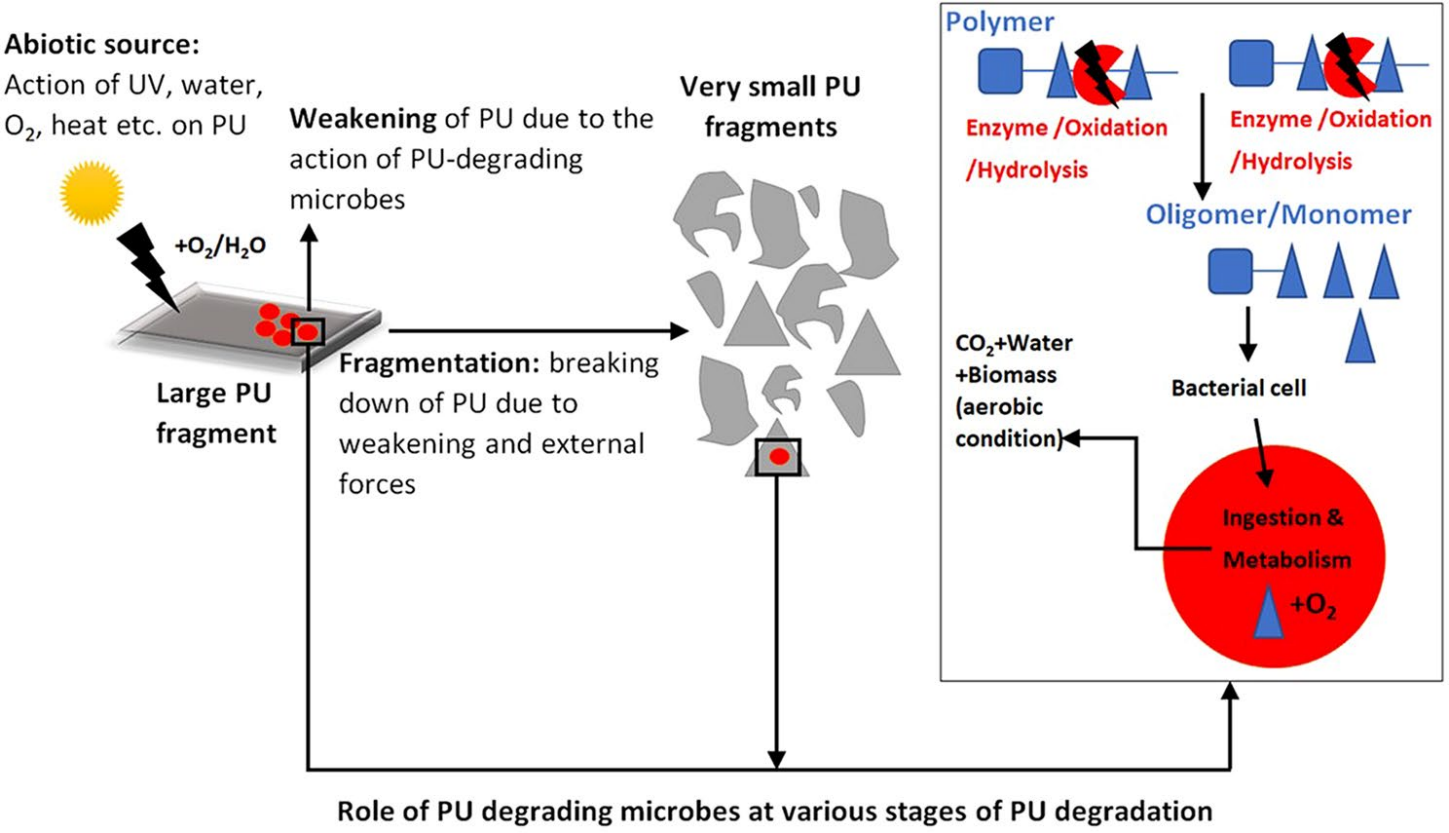
PU degradation in the natural environment. Abiotic factors are major initiators of the degradation process (left); oxidation and/or hydrolysis lead to fragmentation and formation of microplastics. Microbes can adhere to the polymer at any point (left, centre) and further facilitate degradation reactions using enzymes (right). If degradation proceeds to the point of liberation of monomers or sufficiently small oligomers, microbes may internalise said fragments and metabolise them

There are four types of degradation that polymers such as polyurethane can undergo in the environment: photodegradation, thermo-oxidative degradation, hydrolytic cleavage and biodegradation (Andrady 2011). Degradation is commonly kick-started by photodegradation, triggering thermo-oxidative degradation. Hydrolytic cleavage can also occur, further facilitating fragmentation. Microbes in the environment can potentially exert their limited influence at any point (Cosgrove et al. 2007; Andrady 2011), but mineralisation only occurs to a limited degree (Fig. 2). The degree to which each type of degradation occurs largely depends on the environment and the polymer in question, e.g. studies show that polyester polyurethanes are more vulnerable to hydrolysis, whereas polyether and polycarbonate polyurethanes are more vulnerable to oxidation (Stokes et al. 1995; Christenson et al. 2004).

### Photo-oxidative degradation

Ultraviolet light ranges from ~ 295 to 380 nm wavelengths. Bonds such as C–C, C–H and O–H which are commonly found in polymers absorb light of wavelengths below 200 nm, whereas carbonyls and conjugated double bonds absorb light between 200 and 300 nm. Most plastics also contain impurities or additives which absorb UV light. UV radiation excites the electrons in either the polymer or the impurities R*, supplying the energy to transfer them to an acceptor, most commonly O_2_ (I). This causes the formation of RO_2_* free radicals, which react with the polymer (RH), forming hydroperoxide groups (II). This reaction results in the breakage of the polymer chain. Hydroperoxide is unstable under light and increased temperature and breaks down to give two free-radicals RO* and HO* (III), which are then available to continue the process (Feldman 2002). Photo-oxidative degradation occurs preferentially at the material surface due to the low permeability of oxygen and generally results in the yellowing of clear polymers or discolouration of coloured ones, loss of surface shine and surface cracking (Singh et al. 2001; Feldman 2002).

I R*+O_2_→RO_2_*
II RO_2_*+RH→ROOH+R*
III ROOH→RO*+HO*

### Hydrolytic degradation

**Fig. 3:**
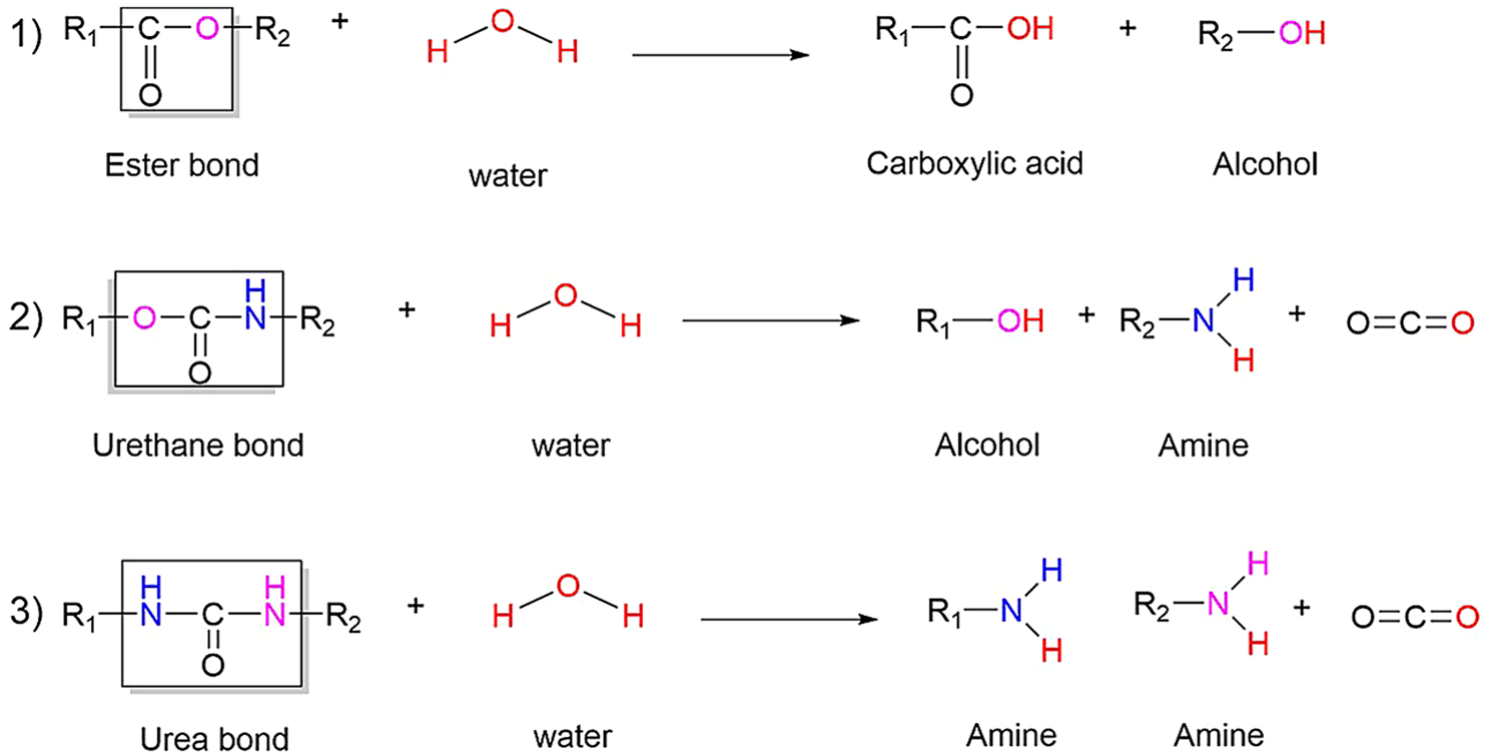
Possible hydrolysis of some types of bonds in PU. Heath and Cooper (2013)

Certain functional groups within the backbone of a polymer can render it susceptible to hydrolysis. Figure 3 shows some possible hydrolytic degradation reactions that can occur in various types of PU. Hydrolysis can occur at ester bonds, urethane bonds and urea bonds present in the polymer. Hydrolysis of ester bonds cleaves the polymer chain, with one of the newly generated free ends terminated by a carboxylic acid group and the other by an alcohol (Fig. 3.1). Hydrolysis of a urethane bond yields an alcohol and an amine, emitting a molecule of CO_2_ in the process (Fig. 3.2). Similarly, hydrolysis of a urea bond yields two amines and the emission of CO_2_ (Fig. 3.3) (Cauich-Rodríguez et al. 2013). Hydrolytic degradation is limited both by the availability of water and the access it has to the relevant functional groups.

### Biodegradation

During biodegradation, the microbes in the environment can use the plastic as a carbon and/or energy source, incorporating carbon liberated from the polymer backbone into its own structures or converting it to carbon dioxide or small organic molecules and thereby generating energy. The biodegradation process can be divided into four stages. The first, biodeterioration, is where biotic factors such as the action of enzymes, production of organic acids, or oxidation processes cause deleterious changes to the polymer, generally starting from the surface and affecting the matrix (Lucas et al. 2008). This is followed by biofragmentation, where the long polymeric chain is cleaved at specific vulnerable sites such as ester groups. This type of action mainly results in the formation of hydrophilic monomers/oligomers (Jin et al. 2021). These compounds are more easily taken up and metabolised by other microbes in the vicinity, leading to “bioassimilation”. The final stage, mineralisation, is where the carbon in the polymer is ultimately converted to cell mass and carbon dioxide.

## Biodegradation of polyurethane

A trend is seen in polyurethane biodegradation in which the amorphous segments of the PU start disintegrating before the crystalline segments (Huang and Roby 1986). The amorphous segments of PU consist of less densely packed polymer chains and contain functional groups such as esters, which are easily hydrolysed (Jin et al. 2021). These regions are more readily accessible to the microbes for biodeterioration, unlike the crystalline regions which are denser and more orderly packed (Kemona and Piotrowska 2020). Biofragmentation can then more readily occur (Ibrahim et al. 2009; Thirunavukarasu et al. 2015), and subsequently greater bioassimilation (Kemona and Piotrowska 2020).

The rate at which polyurethanes biodegrade depends greatly on the material composition and properties. Magnin et al. (2019a) determined how efficiently four thermoplastic PU (TPU) with different macromolecular architectures were degraded by fungi. They found that poly(ester ether)-based TPU and polyether-based TPU showed no significant weight loss over two months, whereas TPU containing polycaprolactone or fatty acid dimers reduced in mass from 1.7 to 9% after two months. Pfohl et al. (2022) examined the effect of hard segment content, presence of hydrolysis stabilisers and cross-linking on degradability. It was found that the extent and the rate of biodegradation decreased as the density of cross-linking and the amount of hard segment increased, and there was less fragmentation of the PU particles in the presence of hydrolysis stabilisers. The greatest extent of biodegradation they observed was 72.3%.

To make biodegradation viable for eliminating PU waste, a thorough understanding of how the organisms biodegrade polyurethane is necessary. Properly facilitated, biodegradation has the potential to achieve complete mineralisation of plastic waste. It can often be a commensal process, requiring different organisms to contribute to each stage of biodegradation to different degrees (Lucas et al. 2008). The main players in the biodegradation of plastics are bacteria and fungi, and in the case of polyurethane degradation fungi dominate. Each has ways, however, of degrading polyurethane with inherent pros and cons (Crabbe et al. 1994; Barratt et al. 2003; Cosgrove et al. 2007).

### Fungal biodegradation

Fungi have higher enzyme diversity and biotic and abiotic stress tolerance than bacteria, and thanks to this they dominate the biodegradation of polyurethane. Although many bacteria can degrade PU, many studies have shown that samples of PU collected from dumping sites were more heavily colonised by fungi than bacteria (Barratt et al. 2003; Cosgrove et al. 2007). Examples exist in the literature of fungi that have been shown to be able to survive using polyurethane as sole carbon and/or energy source (Khruengsai et al. 2022; Liu et al. 2023), however degradation rates can vary significantly by material. For example, Liu et al. (2023) described a strain of *Cladosporium* which could solubilise 32.42% of a poly(butylene adipate)-based material over 28 days, while Magnin et al. (2019a) achieved 8.9% weight loss of a polycaprolactone based material over two months with a strain of *Penicillium*. Fungi are also known to secrete a wide range of substances, including enzymes, into their surroundings (Yang 2006). This is a useful trait for PU biodegradation, because for the bulk polymer to degrade it must come in direct contact with hydrolytic enzymes. Supplementary Table S1 gives a brief list of fungi that have been identified to have PU-degrading potential, and the substrate they were tested on.

In addition to producing hydrolytic enzymes, the mycelia of fungi when growing can exert pressure on the surface of the PU, cracking it and providing a larger exposed surface area for biodegradation to take place (Khan et al. 2017). These mycelia can also grow into the cracks, further weakening the PU in the process. Magnin et al. (2019a) tested three different fungi for their capacity to degrade various PUs, and when samples were viewed by scanning electron microscopy (SEM) after treatment with a strain of *Penicillium* irregular channels on the surface of the PU were observed. It was also observed that the diameter of the *Penicillium* filaments and the width of the channels were very similar. In the same study, a strain of *Alternaria* produced deep holes on the surface, and treatment with a strain of *Aspergillus* made the surface of the PU appear irregular with occasional small cracks (Magnin et al. 2019a).

### Bacterial biodegradation

While bacteria are generally considered less potent than fungi when it comes to degrading polyurethane, many pieces of evidence suggest that they do have good potential to do so. In previous literature the majority of the bacteria that can utilise PU belong to the genera *Bacillus*, *Pseudomonas*, and *Acinetobacter* (Kay et al. 1991; Gautam et al. 2007; Howard et al. 2012; Shah et al. 2013; Nakkabi et al. 2015; Rafiemanzelat et al. 2015; Hung et al. 2016; Vargas-Suárez et al. 2019; Espinosa et al. 2020). Amongst these bacteria, *P. aeruginosa* ATCC 13388 is recommended by the American Society for Testing and Materials (ASTM) for testing whether a polymer is resistant to bacterial degradation (Kay et al. 1991). Some other genera such as *Comamonas* (Nakajima-Kambe et al. 1995), *Micrococcus* (Rafiemanzelat et al. 2015; Vargas-Suárez et al. 2019), *Alicycliphilus* (Pérez-Lara et al. 2016), *Corynebacterium* (Shah et al. 2008) and *Staphylococcus* (Curia et al. 2014) have also been associated with PU biodegradation. Supplementary Table S2 contains a list of bacteria that can degrade PU and the types of PU they are known to be able to degrade.

In a study carried out by Shah et al. (2013) polyester PU film pieces were incubated with a strain of *Pseudomonas aeruginosa* for 4 weeks. When the surfaces were analysed using scanning electron microscopy (SEM), the bacteria were seen attached to the polymer surface with small cracks originating from the point of contact, suggesting that the bacteria had some effect of degradation on the polymer. Rafiemanzelat et al. (2015) showed holes, pits, black spots, erosions, and cracks on the surface of a PU block copolymer after incubation with *Bacillus amyloliquefaciens* M3. A 30–40% reduction in the PUs weight was also observed. FTIR analysis showed that absorption bands corresponding to C=O (urethane, amide, and urea), N–H, C–N, C–O, and C–H changed significantly compared to the control, indicating a change to its chemical backbone. Curia et al. (2014) also demonstrated the formation of micro- and nano-sized particles from bulk PU surfaces by *Staphylococcus aureus*.

Most research focuses on isolating PU degrading microbes from environmental samples *in vitro*, but there is another approach that is proving to have even greater potential, i.e. degradation of PU with the help of insects and their gut microbiome (Liu et al. 2022; Yang et al. 2023). The larvae of *Tenebrio molitor* can grow on a diet purely containing polyether-PU foams, resulting in a 67% weight loss of the PU foam after 35 days. The survival rate of the larvae was similar to those that were bran-feed. The larvae frass contained foam fragments that showed ether and urethane bond scission, indicating partial biodegradation. Through high-throughput sequencing, it was confirmed that there was an increase in the microbial population belonging to the families of *Enterobacteriaceae* and *Streptococcaceae* in the polyether-PU foam-fed larvae (Liu et al. 2022).

### Enzymes involved

A comprehensive mechanism underlying the degradation of polyurethane by microbes is not known, but the enzymes involved have esterase, protease or lipase activity and may be membrane-bound or secreted (Akutsu et al. 1998; Howard and Blake 1998; Howard et al. 2001). A study carried out on an esterase purified from the bacterium *Comamonas acidovorans* showed that the enzyme degrades the PU in two steps: first, binding of the enzyme to the PU surface hydrophobically through the enzyme’s surface-binding domain and second, hydrolysing the ester bonds present via its catalytic domain (Akutsu et al. 1998). This type of action by the membrane-bound enzymes could have advantages over secreted enzymes as it can overcome the hydrophobic nature of many polyurethanes (Barcoto and Rodrigues 2022). In terms of fungal enzymes, only a few studies related to their characterisation have been published to date. Supplementary Table S3 lists some of the enzymes that have been purified, their sources and the PU substrate(s) they were tested upon. For more effective degradation multiple enzymes can work cooperatively; a mixture of esterase and amidase was proven to work synergistically to degrade PU and enable recovery of some of its building blocks (Magnin et al. 2019b). The esterase enzyme performed initial biofragmentation of PU, releasing fragments of low molecular mass containing urethane bonds, which were then further hydrolysed by an amidase.

### Degradation products

Depending on the specificity of the enzyme and the chemical structure of the PU, the degradation products vary. To detect the type of product produced after degradation the products are generally separated by chromatographic techniques (Wang et al. 1997; Shah et al. 2013; Pérez-Lara et al. 2016), with either mass spectrometer (Shah et al. 2013) or UV-visible spectroscope detectors (Thirunavukarasu et al. 2015). The products are not always the original input monomers, e.g. 2,4-toluene diamine (Nakajima-Kambe et al. 1997) and 4,4’-methylene dianiline (Matsumiya et al. 2009; Khan et al. 2017; Magnin et al. 2019b) have been shown to be released from the degradation of a PU produced from 2,4-toluene diisocyanate (TDI) and 4,4′-methylene diphenyl diisocyanate, (MDI), respectively. Other by-products such as adipic acid (Shah et al. 2013; Thirunavukarasu et al. 2015), diethylene glycol (Ibrahim et al. 2009; Thirunavukarasu et al. 2015), 6-hydroxycaproic acid (Magnin et al. 2019b), trimethylolpropane (Ibrahim et al. 2009) and 1,4-butanediol (Shah et al. 2013) have also been detected in other studies.

## Conclusion and future directions

Given the ever-increasing demand for plastics like polyurethane, it is essential to also realise the impacts its waste will have on the environment. Due to the many drawbacks of the conventional methods to dispose of these wastes, a ‘greener’ alternative is necessary, and biodegradation appears to be the most promising option. Biodegradation has the potential to completely mineralise plastic waste, or recover the input materials and better enable recycling. There are hurdles to overcome however, primarily with the efficiency of the process. Inherently different chemical structures of plastic waste further complicate this difficulty. To overcome these limitations, more research needs to be performed not only on improving PU biodegradation but extending what is known to a greater variety of plastics. Future research should aim to find and/or develop new microbes that can degrade multiple different plastics rapidly and efficiently, and to create consortia of plastic-degrading microbes by using the already known plastic degraders. Current progress in the field is encouraging and gives cause to be hopeful that we can soon resolve one of our world’s biggest and most persistent problems.

## Supplementary Information

Below is the link to the electronic supplementary material.

Supplementary material 1 (DOCX 86.9 kb)
