# Supplementary material for: Solving the plastic dilemma: the fungal and bacterial biodegradability of polyurethanes
Source: World J Microbiol Biotechnol. 2023 Mar 17;39(5):122. doi: 10.1007/s11274-023-03558-8 (PMC10020256; doi:10.1007/s11274-023-03558-8)
Supplement: Supplementary file 1 — Supplementary material 1 (DOCX 86.9 kb) [file 11274_2023_3558_MOESM1_ESM.docx]

**Supplemental file**

**Solving the plastic dilemma: The fungal and bacterial biodegradability of polyurethanes**

Parth Bhavsar^a^, Mrinal Bhave^a^, Hayden K. Webb^a,*^

^a^Department of Chemistry and Biotechnology, Swinburne University of Technology, Hawthorn, VICTORIA- 3122, Australia

^*^Corresponding author.

*E-mail address:* [hkwebb@swin.edu.au](mailto:hkwebb@swin.edu.au)

Postal address:

Mail H44, Swinburne University of Technology, Department of Chemistry and Biotechnology, PO Box 218, HAWTHORN, VIC-3122

Some of the discovered fungi that can degrade polyurethane.

| Species/strain | Polyurethane type | References |
| --- | --- | --- |
| *Alternaria* sp. | PU Varnish (Impranil DLN) and thermoplastic polyester PU | [Magnin et al. (2019a)](#_ENREF_29) |
|  | Ether type PU | [Matsumiya et al. (2009)](#_ENREF_32) |
| *Alternaria solani* | Polyester PU |  |
| *Alternaria tenuissima* | Polyether PU based on pyridine | [Oprea et al. (2018)](#_ENREF_38) |
| *Aspergillus flavus* G10 | Polyester PU | [Khan et al. (2017)](#_ENREF_26) |
| *Aspergillus flavus* ITCC 6051 | Polyester PU | [Mathur and Prasad (2012)](#_ENREF_31) |
| *Aspergillus fischeri* | Polyester PU foam | Bentham *et al.* (1987) |
| *Aspergillus fumigatus* | PU varnish (Impranil DLN) and Polyether PU | [Alvarez-Barragan et al. (2016)](#_ENREF_2) |
|  | Hard and soft polyester PU | [Pathirana and Seal (1985)](#_ENREF_39) |
| *Aspergillus niger* | Rigid PU foam based on lignin | [Amaral et al. (2012)](#_ENREF_3) |
|  | PU polyether foam | [Filip (1979)](#_ENREF_16) |
| *Aspergillus* section *Flavi* | PU Varnish (Impranil DLN) and thermoplastic polyester PU | [Magnin et al. (2019a)](#_ENREF_29) |
| *Aspergilus tubingensis* | Polyester PU beads | [Khan et al. (2017)](#_ENREF_26) |
| *Aspergillus versicolor* | Different types of PU | [Darby and Kaplan (1968)](#_ENREF_12) |
| *Aureobasidium pullulans* | PU Varnish (Impranil DLN) | [Crabbe et al. (1994)](#_ENREF_9) |
| *Chaetomium globosum* | Different types of PU | [Darby and Kaplan (1968)](#_ENREF_12) |
|  | PU based on cellulose derivatives and polycaprolactone diol | [Oprea et al. (2019)](#_ENREF_37) |
|  | Hard and soft polyester PU | [Pathirana and Seal (1985)](#_ENREF_39) |
| *Cladosporium asperulatum*  *C. montecillanum*  *C. pseudocladosporioides*  *C. tenuissimum*  *Penicillium chrysogenum* | PU Varnish (Impranil DLN) and Polyether PU | [Alvarez-Barragan et al. (2016)](#_ENREF_2) |
| *Cylindrocladiella parva* | Polyester PU | [Cosgrove et al. (2007)](#_ENREF_8) |
| *Cryptococcus laurentii* | PU Varnish (Impranil DLN) | [Zicht (2017)](#_ENREF_56) |
| *Curvularia senegalensis, Fusarium solani* | PU Varnish (Impranil DLN) | [Crabbe et al. (1994)](#_ENREF_9) |
| *Geomyces pannorum* | Polyester PU | [Cosgrove et al. (2007)](#_ENREF_8) |
| *Gliocladium roseum* | Hard and soft polyester PU | [Pathirana and Seal (1985)](#_ENREF_39) |
| *Papiliotrema laurentii* | Polyester PU coating | [Hung et al. (2019)](#_ENREF_23) |
| *Penicillium citrinum* | Hard and soft polyester PU | [Pathirana and Seal (1985)](#_ENREF_39) |
| *Penicillium* section *Lanata-Divaricata* | PU Varnish (Impranil DLN) and thermoplastic polyester PU | [Magnin et al. (2019a)](#_ENREF_29) |
| *Pestalotiopsis microspora* | PU Varnish (Impranil DLN and DLF) | [Russell et al. (2011)](#_ENREF_46) |
| *Pleurotus ostreatus*, *Trichoderma harzianum* | PU made from polyethylene glycol, methylene diphenyl diisocyanate and Kepok banana hump | [Budirohmi et al. (2020)](#_ENREF_6) |


Bacteria that can degrade polyurethane, and the specific forms they have been shown to degrade.

| Species/strain | Polyurethane type | References |
| --- | --- | --- |
| *Acinetobacter* sp. | Polyester PU coating | [Vargas-Suárez et al. (2019)](#_ENREF_53) |
| *Acinetobacter calcoaceticus*  *Arthrobacter globiformis* | Polyester polyurethane paint | [El-Sayed et al. (1996)](#_ENREF_13) |
| *Acinetobacter gerneri* P7 | PU Varnish (Impranil DLN) | [Howard et al. (2012)](#_ENREF_21) |
| *Alicycliphilus* sp. BQ8 and BQ1 | Polyester PU | [Oceguera-Cervantes et al. (2007)](#_ENREF_36); [Pérez-Lara et al. (2016)](#_ENREF_41) |
| *Bacillus amyloliquefaciens*  *Escherichia coli* | Poly(ether-urethane-urea) based on a α-amino acid | [Rafiemanzelat et al. (2015)](#_ENREF_43) |
| *Bacillus subtilis* MZA-75 | Polyester PU | [Shah et al. (2013)](#_ENREF_49) |
| *Bacillus subtilis* | PU Varnish (Impranil DLN) | [Nakkabi et al. (2015)](#_ENREF_34) |
| *Bacillus* sp. | Polyester PU coating | [Vargas-Suárez et al. (2019)](#_ENREF_53) |
|  | Polyester PU | [Shah et al. (2008)](#_ENREF_48) |
| *Comamonas acidovorans* | Polyester PU | [Nakajima-Kambe et al. (1995)](#_ENREF_33); [Akutsu et al. (1998)](#_ENREF_1); [Nomura et al. (1998)](#_ENREF_35) |
|  | Polyether PU | [Nakajima-Kambe et al. (1995)](#_ENREF_33) |
| *Corynebacterium* sp. | PU polyester foam | [Kay et al. (1991)](#_ENREF_25) |
|  | Polyester PU | [Howard and Blake (1998)](#_ENREF_19); [Shah et al. (2008)](#_ENREF_48) |
| *Chryseobacterium meningosepticum* | PU polyester foam | [Cangemi et al. (2008)](#_ENREF_7) |
| *Microbacterium* sp. | Polyester PU coating | [Vargas-Suárez et al. (2019)](#_ENREF_53) |
| *Micrococcus* sp. | Poly(ether-urethane-urea) based on a α-amino acid | [Rafiemanzelat et al. (2013)](#_ENREF_42) |
|  | Polyester PU | [Shah et al. (2008)](#_ENREF_48) |
| *Pseudomonas aeruginosa* ATCC 13388 | PU polyester foam | [Kay et al. (1991)](#_ENREF_25) |
| *Pseudomonas aeruginosa* MZA-85 | Polyester PU | [Shah et al. (2013)](#_ENREF_49) |
| *Pseudomonas chlororaphis* | PU Varnish (Impranil DLN) | [Howard et al. (1999)](#_ENREF_22) |
| *Pseudomonas chlororaphis* ATCC55729 | PU polyester foam | [Gautam et al. (2007)](#_ENREF_18) |
| *Pseudomonas fluorescens* | PU Varnish (Impranil DLN) | [Howard and Blake (1998)](#_ENREF_19) |
|  | Polyether PU coating | [Crookes-Goodson et al. (2013)](#_ENREF_10) |
| *Pseudomonas protegens* pf-5 | PU Varnish (Impranil DLN) | [Biffinger et al. (2014)](#_ENREF_5); [Hung et al. (2016)](#_ENREF_24) |
| *Pseudomonas putida* | PU Varnish (Impranil DLN) | [Peng et al. (2014)](#_ENREF_40) |
| *Pseudomonas* sp. TDA_1_ | PU precursor 2,4 TDA and Oligomer of PU | [Espinosa et al. (2020)](#_ENREF_14) |
| *Staphylococcus aureus* | Poly(ether-urethane-urea) based on a α-amino acid | [Rafiemanzelat et al. (2013)](#_ENREF_42) |
|  | PU with a variety of surface finishes. | [Curia et al. (2014)](#_ENREF_11) |


Microbial enzymes able to degrade PU.

| Class | Enzyme name | PU substrate | References |
| --- | --- | --- | --- |
| Amidase | *Escherichia coli* E4143 Amidase | Polyester PU | [Magnin et al. (2019b)](#_ENREF_30) |
|  | *Nocardia farcinica* polyamidase | Polyester PU | [Gamerith et al. (2016)](#_ENREF_17) |
| Esterase | *Bacillus subtilis* esterase | PU Varnish (Impranil DLN) | [Rowe and Howard (2002)](#_ENREF_44) |
|  | *Comamonas acidovorans* TB-35 esterase (pudA) | PU Varnish (Impranil DLN) | [Akutsu et al. (1998)](#_ENREF_1); [Nomura et al. (1998)](#_ENREF_35) |
|  | *Curvularia senegalensis* esterase | PU Varnish (Impranil DLN) | [Crabbe et al. (1994)](#_ENREF_9) |
|  | *Escherichia coli* esterase (E3576) | PU Varnish (Impranil DLN); Polyester PU | [Magnin et al. (2019b)](#_ENREF_30) |
|  | *Pseudomonas fluorescens* esterase; gene:pulA | PU Varnish (Impranil DLN) | [Ruiz and Howard (1999)](#_ENREF_45); [Biffinger et al. (2015)](#_ENREF_4) |
|  | *Pseudomonas chlororaphis* esterase | PU Varnish (Impranil DLN) | [Howard et al. (1999)](#_ENREF_22) |
| Hydrolase | *Thermomonospora curvata* DSM43183 (Tcur0390)  *Thermomonospora curvata* DSM43183 (Tcur1278) | PU Varnish (Impranil DLN); Polyester PU | [Schmidt et al. (2017)](#_ENREF_47) |
| Lipase | *Cryptococcus* sp. MTCC 5455 lipase | Polyester PU | [Thirunavukarasu et al. (2015)](#_ENREF_52) |
|  | *Pseudomonas fluorescens* lipase AK | pH-sensitive biodegradable PCL-LDI based PU | [Zhou et al. (2012)](#_ENREF_54) |
|  | *Pseudomonas cepacian* lipase | Poly (ester ether) PU | [Zhou and Xie (2017)](#_ENREF_55) |
|  | *Pseudomonas* sp. lipase | PU Varnish (Impranil DLN) | [Biffinger et al. (2015)](#_ENREF_4) |
|  | *Pseudomonas chlororaphis* pueA | PU Varnish (Impranil DLN) | [Stern and Howard (2000)](#_ENREF_50) |
|  | *Pseudomonas chlororaphis* pueB | PU Varnish (Impranil DLN) | [Howard et al. (2001)](#_ENREF_20) |
|  | *Thermomyces lanuginosus* lipase | Poly (ester urea) PU | [Fang et al. (2014)](#_ENREF_15) |
|  | *Candida antarctica* lipase | Polyester PU | [Takamoto et al. (2001)](#_ENREF_51) |
|  | *Candida cylindracea* lipase | Polyester PU | [Kim and Kim (1998)](#_ENREF_27) |
|  | *Candida rugosa* lipase | Polyester PU | [Li et al. (2015)](#_ENREF_28) |
|  | *Thermobifida fusca* KW3 cutinase (TfCut 2) | PU Varnish (Impranil DLN); Polyester PU | [Schmidt et al. (2017)](#_ENREF_47) |
| Protease | *Bacillus* sp. Protease | PU Varnish (Impranil DLN) | [Biffinger et al. (2015)](#_ENREF_4) |
|  | *Pseudomonas fluorescens* protease | PU Varnish (Impranil DLN) | [Howard and Blake (1998)](#_ENREF_19) |

Akutsu Y, Nakajima-Kambe T, Nomura N, Nakahara T (1998) Purification and properties of a polyester polyurethane-degrading enzyme from *Comamonas acidovorans* TB-35. Appl. Environ. Microbiol. 64(1): 62. <https://doi.org/10.1128/aem.64.1.62-67.1998>

Alvarez-Barragan J, Dominguez-Malfavon L, Vargas-Suarez M, Gonzalez-Hernandez R, Aguilar-Osorio G, Loza-Tavera H (2016) Biodegradative activities of selected environmental fungi on a polyester polyurethane varnish and polyether polyurethane foams. Appl. Environ. Microbiol. 82(17): 5225-5235. <https://doi.org/10.1128/AEM.01344-16>

Amaral JS, Sepúlveda M, Cateto CA, Fernandes IP, Rodrigues AE, Belgacem MN, Barreiro MF (2012) Fungal degradation of lignin-based rigid polyurethane foams. Polym. Degrad. Stab. 97(10): 2069-2076. <https://doi.org/10.1016/j.polymdegradstab.2012.03.037>

Biffinger JC, Barlow DE, Cockrell AL, Cusick KD, Hervey WJ, Fitzgerald LA, Nadeau LJ, Hung CS, Crookes-Goodson WJ, Russell JN (2015) The applicability of Impranil®DLN for gauging the biodegradation of polyurethanes. Polym. Degrad. Stab. 120: 178-185. <https://doi.org/10.1016/j.polymdegradstab.2015.06.020>

Biffinger JC, Barlow DE, Pirlo RK, Babson DM, Fitzgerald LA, Zingarelli S, Nadeau LJ, Crookes-Goodson WJ, Russell JN (2014) A direct quantitative agar-plate based assay for analysis of *Pseudomonas protegens* Pf-5 degradation of polyurethane films. Polym. Degrad. Stab. 95: 311-319. <https://doi.org/10.1016/j.ibiod.2014.09.005>

Budirohmi A, Ahmad A, Taba P (2020) Characterization and TiO_2_-catalyzed degradation of polyurethane biopolymer through medium pro compost. Orient. J. Chem. 36(1): 161-165. <https://doi.org/10.13005/ojc/360121>

Cangemi JM, Dos Santos AM, Neto SC, Chierice GO (2008) Biodegradation of polyurethane derived from castor oil. Polimeros 18(3). <https://doi.org/10.1590/s0104-14282008000300004>

Cosgrove L, McGeechan PL, Robson GD, Handley PS (2007) Fungal communities associated with degradation of polyester polyurethane in soil. Appl. Environ. Microbiol. 73(18): 5817. <https://doi.org/10.1128/AEM.01083-07>

Crabbe JR, Campbell JR, Thompson L, Walz SL, Schultz WW (1994) Biodegradation of a colloidal ester-based polyurethane by soil fungi. Int. Biodeterior. Biodegrad. 33(2): 103-113. <https://doi.org/10.1016/0964-8305(94)90030-2>

Crookes-Goodson WJ, Bojanowski CL, Kay ML, Lloyd PF, Blankemeier A, Hurtubise JM, Singh KM, Barlow DE, Ladouceur HD, Matt Eby D, Johnson GR, Mirau PA, Pehrsson PE, Fraser HL, Russell Jr JN (2013) The impact of culture medium on the development and physiology of biofilms of *Pseudomonas fluorescens* formed on polyurethane paint. Biofouling 29(6): 601-615. <https://doi.org/10.1080/08927014.2013.783906>

Curia R, Milani M, Didenko L, Avtandilov G, Shevlyagina N, Smirnova T 2014 Beyond the biodestruction of polyurethane: *S. aureus* uptake of nanoparticles is a challenge for toxicology. In: Microsc. Adv. Sci. Res. Educ. Méndez-Vilas A**:** 16-23.

Darby RT, Kaplan AM (1968) Fungal susceptibility of polyurethanes. Appl Microbiol 16(6): 900-905. <https://doi.org/10.1128/am.16.6.900-905.1968>

El-Sayed AHMM, Mahmoud WM, Davis EM, Coughlin RW (1996) Biodegradation of polyurethane coatings by hydrocarbon-degrading bacteria. Int. Biodeterior. Biodegrad. 37(1-2): 69-79. <https://doi.org/10.1016/0964-8305(95)00091-7>

Espinosa MJC, Blanco AC, Schmidgall T, Atanasoff-Kardjalieff AK, Kappelmeyer U, Tischler D, Pieper DH, Heipieper HJ, Eberlein C (2020) Toward biorecycling: Isolation of a soil bacterium that grows on a polyurethane oligomer and monomer. Frontiers Microbiol. 11. <https://doi.org/10.3389/fmicb.2020.00404>

Fang J, Ye S-H, Shankarraman V, Huang Y, Mo X, Wagner WR (2014) Biodegradable poly(ester urethane)urea elastomers with variable amino content for subsequent functionalization with phosphorylcholine. Acta Biomater. 10(11): 4639-4649. <https://doi.org/10.1016/j.actbio.2014.08.008>

Filip Z (1979) Polyurethane as the sole nutrient source for *Aspergillus niger* and *Cladosporium herbarum*. Eur. J. Appl. Microbiol. Biotechnol. 7(3): 277-280. <https://doi.org/10.1007/bf00498022>

Gamerith C, Herrero Acero E, Pellis A, Ortner A, Vielnascher R, Luschnig D, Zartl B, Haernvall K, Zitzenbacher S, Strohmeier G, Hoff O, Steinkellner G, Gruber K, Ribitsch D, Guebitz GM (2016) Improving enzymatic polyurethane hydrolysis by tuning enzyme sorption. Polym. Degrad. Stab. 132: 69-77. <https://doi.org/10.1016/j.polymdegradstab.2016.02.025>

Gautam R, Bassi AS, Yanful EK, Cullen E (2007) Biodegradation of automotive waste polyester polyurethane foam using *Pseudomonas chlororaphis* ATCC55729. Int. Biodeterior. Biodegrad. 60(4): 245-249. <https://doi.org/10.1016/j.ibiod.2007.03.009>

Howard GT, Blake RC (1998) Growth of *Pseudomonas fluorescens* on a polyester–polyurethane and the purification and characterization of a polyurethanase–protease enzyme. Int. Biodeterior. Biodegrad. 42(4): 213-220. <https://doi.org/10.1016/S0964-8305(98)00051-1>

Howard GT, Crother B, Vicknair J (2001) Cloning, nucleotide sequencing and characterization of a polyurethanase gene (pueB) from *Pseudomonas chlororaphis*. Int. Biodeterior. Biodegrad. 47(3): 141-149. <https://doi.org/10.1016/S0964-8305(01)00042-7>

Howard GT, Norton WN, Burks T (2012) Growth of *Acinetobacter gerneri* P7 on polyurethane and the purification and characterization of a polyurethanase enzyme. Biodegradation 23(4): 561-573. <https://doi.org/10.1007/s10532-011-9533-6>

Howard GT, Ruiz C, Hilliard NP (1999) Growth of *Pseudomonas chlororaphis* on a polyester–polyurethane and the purification andcharacterization of a polyurethanase–esterase enzyme. Int. Biodeterior. Biodegrad. 43(1): 7-12. <https://doi.org/10.1016/S0964-8305(98)00057-2>

Hung C-S, Barlow DE, Varaljay VA, Drake CA, Crouch AL, Russell JN, Nadeau LJ, Crookes-Goodson WJ, Biffinger JC (2019) The biodegradation of polyester and polyester polyurethane coatings using *Papiliotrema laurentii*. Int. Biodeterior. Biodegrad. 139: 34-43. <https://doi.org/10.1016/j.ibiod.2019.02.002>

Hung C-S, Zingarelli S, Nadeau LJ, Biffinger JC, Drake CA, Crouch AL, Barlow DE, Russell Jr JN, Crookes-Goodson WJ (2016) Carbon catabolite repression and Impranil polyurethane degradation in *Pseudomonas protegens* strain Pf-5. Appl. Environ. Microbiol. 82(20): 6080. <https://doi.org/10.1128/AEM.01448-16>

Kay MJ, Morton LHG, Prince EL (1991) Bacterial degradation of polyester polyurethane. Int. Biodeterior. 27(2): 205-222. <https://doi.org/10.1016/0265-3036(91)90012-G>

Khan S, Nadir S, Shah ZU, Shah AA, Karunarathna SC, Xu J, Khan A, Munir S, Hasan F (2017) Biodegradation of polyester polyurethane by *Aspergillus tubingensis*. Environ. Pollut. 225: 469-480. <https://doi.org/10.1016/j.envpol.2017.03.012>

Kim YD, Kim SC (1998) Effect of chemical structure on the biodegradation of polyurethanes under composting conditions. Polym. Degrad. Stab. 62(2): 343-352. <https://doi.org/10.1016/S0141-3910(98)00017-2>

Li S-L, Wu F, Wang Y-Z, Zeng J-B (2015) Biobased thermoplastic poly(ester urethane) elastomers consisting of poly(butylene succinate) and poly(propylene succinate). Ind. Eng. Chem. Res. 54(24): 6258-6268. <https://doi.org/10.1021/acs.iecr.5b00637>

Magnin A, Hoornaert L, Pollet E, Laurichesse S, Phalip V, Avérous L (2019a) Isolation and characterization of different promising fungi for biological waste management of polyurethanes. Microb. Biotechnol. 12(3): 544-555. <https://doi.org/10.1111/1751-7915.13346>

Magnin A, Pollet E, Perrin R, Ullmann C, Persillon C, Phalip V, Avérous L (2019b) Enzymatic recycling of thermoplastic polyurethanes: Synergistic effect of an esterase and an amidase and recovery of building blocks. Waste Manag. 85: 141-150. <https://doi.org/10.1016/j.wasman.2018.12.024>

Mathur G, Prasad R (2012) Degradation of polyurethane by *Aspergillus flavus* (ITCC 6051) isolated from soil. Appl. Biochem. Biotechnol. 167(6): 1595-1602. <https://doi.org/10.1007/s12010-012-9572-4>

Matsumiya Y, Murata N, Tanabe E, Kubota K, Kubo M (2009) Isolation and characterization of an ether-type polyurethane-degrading micro-organism and analysis of degradation mechanism by *Alternaria* sp. J. Appl. Microbiol. <https://doi.org/10.1111/j.1365-2672.2009.04600.x>

Nakajima-Kambe T, Onuma F, Kimpara N, Nakahara T (1995) Isolation and characterization of a bacterium which utilizes polyester polyurethane as a sole carbon and nitrogen source. FEMS Microbiol. Lett. 129(1): 39-42. <https://doi.org/10.1016/0378-1097(95)00131-N>

Nakkabi A, Sadiki M, Fahim M, Ittobane N, Ibnsouda Koraichi S, Barkai H (2015) Biodegradation of poly(ester urethane)s by *Bacillus subtilis*. Int. J. Environ. Res. 9(1): 157-162. <https://doi.org/10.22059/IJER.2015.885>

Nomura N, Shigeno-Akutsu Y, Nakajima-Kambe T, Nakahara T (1998) Cloning and sequence analysis of a polyurethane esterase of *Comamonas acidovorans* TB-35. J. Ferment. Bioeng. 86(4): 339-345. <https://doi.org/10.1016/S0922-338X(99)89001-1>

Oceguera-Cervantes A, Carrillo-Garcia A, Lopez N, Bolanos-Nunez S, Cruz-Gomez MJ, Wacher C, Loza-Tavera H (2007) Characterization of the polyurethanolytic activity of two *Alicycliphilus* sp. strains able to degrade polyurethane and N-methylpyrrolidone. Appl. Environ. Microbiol. 73(19): 6214-6223. <https://doi.org/10.1128/aem.01230-07>

Oprea S, Gradinariu P, Oprea V (2019) Properties and fungal biodegradation of the different cellulose derivatives structure included into castor oil-based polyurethane composites. J. Compos. Mater. 53(25): 3535-3548. <https://doi.org/10.1177/0021998319843334>

Oprea S, Potolinca VO, Gradinariu P, Oprea V (2018) Biodegradation of pyridine-based polyether polyurethanes by the *Alternaria tenuissima* fungus. J. Appl. Polym. Sci. 135(14): 46096. <https://doi.org/10.1002/app.46096>

Pathirana R, Seal K (1985) Studies on polyurethane deteriorating fungi. 3. Physico-mechanical and weight changes during fungal deterioration. Int. Biodeterior. 21(1): 41-49

Peng Y-H, Shih Y-h, Lai Y-C, Liu Y-Z, Liu Y-T, Lin N-C (2014) Degradation of polyurethane by bacterium isolated from soil and assessment of polyurethanolytic activity of a Pseudomonas putida strain. Environ. Sci. Pollut. Res. 21(16): 9529-9537. <https://doi.org/10.1007/s11356-014-2647-8>

Pérez-Lara LF, Vargas-Suárez M, López-Castillo NN, Cruz-Gómez MJ, Loza-Tavera H (2016) Preliminary study on the biodegradation of adipate/phthalate polyester polyurethanes of commercial-type by *Alicycliphilus* sp. BQ8. J. Appl. Polym. Sci. 133(6): n/a-n/a. <https://doi.org/10.1002/app.42992>

Rafiemanzelat F, Fathollahi Zonouz A, Emtiazi G (2013) Synthesis of new poly(ether–urethane–urea)s based on amino acid cyclopeptide and PEG: study of their environmental degradation. Amino Acids 44(2): 449-459. <https://doi.org/10.1007/s00726-012-1353-4>

Rafiemanzelat F, Jafari M, Emtiazi G (2015) Study of biological degradation of new poly(ether-urethane-urea)s containing cyclopeptide moiety and PEG by *Bacillus amyloliquefaciens* isolated from soil. Appl. Biochem. Biotechnol. 177(4): 842-860. <https://doi.org/10.1007/s12010-015-1782-0>

Rowe L, Howard GT (2002) Growth of *Bacillus subtilis* on polyurethane and the purification and characterization of a polyurethanase-lipase enzyme. Int. Biodeterior. Biodegrad. 50(1): 33-40. <https://doi.org/10.1016/S0964-8305(02)00047-1>

Ruiz C, Howard GT (1999) Nucleotide sequencing of a polyurethanase gene (pulA) from *Pseudomonas fluorescens*. Int. Biodeterior. Biodegrad. 44(2-3): 127-131. <https://doi.org/10.1016/s0964-8305(99)00074-8>

Russell JR, Huang J, Anand P, Kucera K, Sandoval AG, Dantzler KW, Hickman D, Jee J, Kimovec FM, Koppstein D, Marks DH, Mittermiller PA, Núñez SJ, Santiago M, Townes MA, Vishnevetsky M, Williams NE, Vargas MPN, Boulanger L-A, Bascom-Slack C, Strobel SA (2011) Biodegradation of polyester polyurethane by endophytic fungi. Appl. Environ. Microbiol. 77(17): 6076. <https://doi.org/10.1128/AEM.00521-11>

Schmidt J, Wei R, Oeser T, Dedavid E Silva L, Breite D, Schulze A, Zimmermann W (2017) Degradation of polyester polyurethane by bacterial polyester hydrolases. Polymers 9(12): 65. <https://doi.org/10.3390/polym9020065>

Shah AA, Hasan F, Akhter JI, Hameed A, Ahmed S (2008) Degradation of polyurethane by novel bacterial consortium isolated from soil. Ann. Microbiol. 58(3): 381. <https://doi.org/10.1007/BF03175532>

Shah Z, Hasan F, Krumholz L, Aktas DF, Shah AA (2013) Degradation of polyester polyurethane by newly isolated *Pseudomonas aeruginosa* strain MZA-85 and analysis of degradation products by GC–MS. Int. Biodeterior. Biodegrad. 77: 114-122. <https://doi.org/10.1016/j.ibiod.2012.11.009>

Stern RV, Howard GT (2000) The polyester polyurethanase gene (pueA) from *Pseudomonas chlororaphis* encodes a lipase. FEMS Microbiol. Lett. 185(2): 163-168. <https://doi.org/10.1111/j.1574-6968.2000.tb09056.x>

Takamoto T, Shirasaka H, Uyama H, Kobayashi S (2001) Lipase-catalyzed hydrolytic degradation of polyurethane in organic solvent. Chem. Lett. 30(6): 492-493. <https://doi.org/10.1246/cl.2001.492>

Thirunavukarasu K, Purushothaman S, Gowthaman MK, Nakajima-Kambe T, Rose C, Kamini NR (2015) Utilization of fish meal and fish oil for production of *Cryptococcus* sp. MTCC 5455 lipase and hydrolysis of polyurethane thereof. J. Food Sci. Technol. 52(9): 5772-5780. <https://doi.org/10.1007/s13197-014-1697-8>

Vargas-Suárez M, Fernández-Cruz V, Loza-Tavera H (2019) Biodegradation of polyacrylic and polyester polyurethane coatings by enriched microbial communities. Appl. Microbiol. Biotechnol. 103(7): 3225-3236. <https://doi.org/10.1007/s00253-019-09660-y>

Zhou L, Liang D, He X, Li J, Tan H, Li J, Fu Q, Gu Q (2012) The degradation and biocompatibility of pH-sensitive biodegradable polyurethanes for intracellular multifunctional antitumor drug delivery. Biomaterials 33(9): 2734-2745. <https://doi.org/10.1016/j.biomaterials.2011.11.009>

Zhou X-M, Xie W-J (2017) Synthesis and characterization of poly(ester ether urethane)s block copolymers based on biodegradable poly(butylene succinate) and poly(ethylene glycol). Polym. Degrad. Stab. 140: 147-155. <https://doi.org/10.1016/j.polymdegradstab.2017.04.023>

Zicht TJ 2017 Detection and analysis of polyurethane biodegradation due to *Cryptococcus laurentii*, Wright State University.
